# Eating disorders and their relationship with menopausal phases among a sample of middle-aged Lebanese women

**DOI:** 10.1186/s12905-022-01738-6

**Published:** 2022-05-10

**Authors:** Joe Khalil, Sarah Boutros, Nelly Kheir, Maha Kassem, Pascale Salameh, Hala Sacre, Marwan Akel, Sahar Obeid, Souheil Hallit

**Affiliations:** 1 School of Medicine and Medical Sciences, Holy Spirit University of Kaslik, P.O. Box 446, Jounieh, Lebanon; 2 Research Department, Psychiatric Hospital of the Cross, P.O. Box 60096, Jal Eddib, Lebanon; 3 Faculty of Medicine, New Vision University, Tbilisi, Georgia; 4 School of Medicine, Lebanese American University, Byblos, Lebanon; 5 Institut National de Santé Publique, Epidémiologie Clinique et Toxicologie (INSPECT-LB), Beirut, Lebanon; 6 Department of Primary Care and Population Health, University of Nicosia Medical School, 2417 Nicosia, Cyprus; 7 Faculty of Pharmacy, Lebanese University, Hadat, Lebanon; 8 School of Pharmacy, Lebanese International University, Beirut, Lebanon; 9 School of Arts and Sciences, Social and Education Sciences Department, Lebanese American University, Jbeil, Lebanon; 10 Psychology Department, College of Humanities, Effat University, Jeddah, 21478 Saudi Arabia

**Keywords:** Binge eating, Orthorexia nervosa, Restrained eating, Menopause, Body dissatisfaction, Lebanon

## Abstract

**Objectives:**

The objective of our study was to evaluate the association between the transition to menopause, body dissatisfaction, and abnormal eating habits (restrained eating, binge eating, and orthorexia nervosa) in a sample of middle-aged Lebanese women.

**Methods:**

A cross-sectional study conducted between July 2019 and January 2020 enrolled 1001 women aged 40 years and above from all Lebanese governorates. Data were collected by face-to-face interviews with all participants. The Body dissatisfaction subscale of the Eating Disorder Inventory‑second version was used to assess body dissatisfaction, whereas the Binge Eating Scale, Dutch Restrained Eating Scale, ORTO-15, Dusseldorf Orthorexia Scale (DOS), and Teruel Orthorexia Scale (TOS) were used to assess eating disorders (binge eating, restrained eating and orthorexia nervosa respectively. Multivariable analysis of covariance (MANCOVA) was used to compare multiple measures among the three menopausal phases, after adjustment over potential confounding variables (age, monthly income, body mass index, marital status, education level, and body dissatisfaction).

**Results:**

Postmenopause was significantly associated with more orthorexia nervosa tendencies (lower ORTO-15 scores) than premenopause (β = − 1.87; *p* = 0.022). Perimenopause was associated with more binge eating (β = 1.56; *p* = 0.031), and less orthorexia nervosa (as measured by the DOS) than premenopause, with this association tending to significance (β  = − 1.10; *p* = 0.051). Furthermore, higher body dissatisfaction was significantly associated with higher restrained eating (β = 0.02; *p* < 0.001), binge eating (β = 0.48; *p* < 0.001), and orthorexia nervosa as measured by ORTO-15 (*β* = − 0.17; *p* < 0.001) and TOS (β = 0.08; *p* = 0.002), but not DOS.

**Conclusion:**

Our study showed that menopausal stages are associated with some disordered eating behaviors (binge eating and orthorexia nervosa) among middle-aged women. Those results may serve as a first step towards spreading awareness among women within this age group regarding eating attitudes. Moreover, healthcare professionals should screen for the presence of disordered eating during those women’s routine visits to the clinics.

**Supplementary Information:**

The online version contains supplementary material available at 10.1186/s12905-022-01738-6.

## Background

Eating disorders (EDs) are characterized by severe and persistent disturbed eating behaviors and associated distressing thoughts and emotions. Sometimes related to body dissatisfaction [1], they are often correlated with an obsession with food, weight, or shape, or anxiety about the consequences of eating certain foods. EDs include several types of disorders [1].

Restrained eating is the intention to limit food consumption on purpose to lose weight, particularly avoiding fatty foods and the use of artificial sweeteners and other calorie-reduced foodstuffs [2]. Binge eating disorder includes the repeated occurrence of overeating episodes, in which people eat enormous amounts of food in a short period, undergo a sense of loss of control over their eating, and are anxious and bothered by this behavior. Binging happens without appropriate compensatory behaviors to eliminate the consumed food [3]. Orthorexia nervosa is a disorder characterized by an obsession with eating healthy food. The term derives from the Greek word *orthos* that means “right” [4]. For people with orthorexia, eating healthy food becomes a risky, obsessive, psychologically limiting, and sometimes physically damaging behavior, as they repeatedly fight against sentiments of being unclean because of the food they have eaten, no matter how delicately they keep track of their diet [5].

Body dissatisfaction is the negative perception of someone’s own body. It is associated with the negative assessment of body size, shape, muscularity, and weight and often encompasses a perceived discordance between the judgment of own body and perfect body [6]. Body dissatisfaction has been previously shown to be associated with more disordered eating (restrained eating, binge eating, and orthorexia nervosa) [7–10] and lower quality of life [11].

Furthermore, studies about disordered eating have been focusing on adolescents/young women; nevertheless, this does not mean that middle-aged women in the final stages of reproductive life are protected from such problems. In fact, about 4.6% of women between 40 and 60 have full-threshold Diagnostic and Statistical Manual of Mental Disorders- 4th edition (DSM-4) norms for an eating disorder, and 4.8% exhibit sub-threshold eating disorder manifestations [12]. One particular feature that is common among middle-aged women and can be a potential reason for an eating disorder is menopause [13].

By definition, menopause is the absence of menses for 12 consecutive months without a pathological cause [6]*.* According to the WHO, and the STRAW classification it is subdivided into three phases: premenopause, perimenopause, and postmenopause. Premenopause is the period that occurs before menopause. During this phase, women continue to menstruate regularly throughout the past 12 months, with no changes in their usual pattern [14, 15]. Perimenopause is the transition period before menopause, described by irregular menses (e.g., changes in the flow, length, or intensity); it lasts for a minimum of three months but no more than 12 months. It is associated with hormonal changes that can lead to increased body weight and fat mass and a redistribution of body fat from the lower body (i.e., hips) to the upper body [14, 15]. Postmenopause is the physiological period following menopause, during which menses are absent for more than 12 consecutive months, not related to medical or surgical interventions [14, 15].

To our knowledge, no studies have yet evaluated the association between orthorexia nervosa and menopausal phases. Thus, there is a need to explore this area since women during these phases tend to be more anxious about their body image [16] because they fear menopause consequences, such as obesity and other health problems [17], and tend to follow healthier behaviors to the extent of reaching orthorexia nervosa. Moreover, previous findings exhibit several discrepancies in terms of disordered eating, likely caused by the way women were grouped, their menopausal phase, and the status of middle-aged women enrolled (with or without kids, married or not) [14]. Some studies suggest that women in their postmenopausal phases reported higher restrained eating and dietary disinhibition than premenopausal women [18]. A study found that disordered eating symptoms were similar among women during menopause (perimenopause and postmenopause) compared to premenopausal women [19]. However, another study indicated that perimenopausal women have a higher incidence of disordered eating, feeling fat, and preoccupation with body shape and weight than premenopausal women [20]. Another study claims no difference between pre, peri, and postmenopausal women concerning disordered eating and body shape concerns [14]. Studies about eating disorders in Lebanon were conducted among the general adult population [21–27] and adolescents [28–30], but not on middle-aged women in their menopause phase. The results of international studies are controversial about disordered eating in general and are lacking about orthorexia nervosa in particular. Consequently, it was deemed necessary to highlight such associations in Lebanon. Therefore, the objective of our study is to assess whether menopause phases are associated with disordered eating (restrained eating, binge eating, and orthorexia nervosa) after adjustment over sociodemographic variables and body dissatisfaction in a sample of middle-aged Lebanese women.

## Methods

### Study design and sampling

This cross-sectional study was conducted between July 2019 and January 2020, among a proportionate sample of women aged 40 years and above recruited from all Lebanese districts. Inclusion criteria were female sex and being aged 40 years and more. No exclusion criteria were applied. Each district was divided into sub-districts, categorized into towns. Within every community, a field worker that was trained for this project by members of the research group, had to interview participants fulfilling the inclusion criteria during the whole period of the study at a place designed by the participant. Participants were arbitrarily designated from each household. Participation was voluntary and participants received no incentive for joining the study. Women who refused to complete the questionnaire were excluded. The method followed was applied in previous papers [31–33].

### Sample size calculation

The G-power 3.1.9.2 software computed a minimum sample of 862 women needed for our study, considering a power of 90%, a proportion p2 = 0.05, and a mean EAT-26 score of 7.51 ± 6.58 in perimenopausal women and 6.29 ± 6.17 in menopausal women, according to Thompson and Bardone-Cone [14]. Out of 1300 questionnaires, a total of 1001 (77%) was completed and collected back.

### Questionnaire

Well-trained pharmacy students collected the data through face-to-face interviews, using a questionnaire in Arabic, the native language in Lebanon. They had received thorough training prior to starting data collection to ensure uniformity.

The questionnaire consisted of two parts. The first one tackled the sociodemographic features of participants: age, gender, marital status, education level, monthly income, and self-reported weight (kg) and height (cm) to calculate the body mass index (BMI). BMI was subdivided into 3 categories (normal: BMI < 25 kg/m^2^, overweight: BMI between 25 and 29.99 kg/m^2^ and obese: BMI of 30 kg/m^2^ or more). Income was divided into four categories: no income, low (< 1000 USD), intermediate (between 1000 and 2000 USD), and high (more than 2000 USD).

The second part of the questionnaire included the following scales.

#### Body dissatisfaction subscale of the eating disorder inventory‑second version (EDI‑2)

The body dissatisfaction subscale evaluates the degree of dissatisfaction with the overall body and particular body parts. It consists of nine items rated on a 4-point Likert scale from never (0) to always (3). The higher the scores, the higher the level of body dissatisfaction [34] (Cronbach’s α = 0.812).

#### Binge Eating Scale (BES)

The BES, validated in Lebanon [8, 28], is a self-report instrument, where participants choose the declaration that suits them most [35]. This 16-item scale determines binge eating symptomatology. The total score is calculated by summing the 16 items, with higher scores indicating more binge eating. Scores < 17 reflect no to mild BE, whereas scores between 18 and 26 and 27 and above indicate moderate and severe BE respectively [36] (Cronbach’s α = 0.897).

#### Dutch Restrained Eating Scale

The Dutch Restrained Eating Scale, validated in Lebanon [7, 37], is a 10-item tool used to measure the recurrence of dieting behaviors on a 5-point Likert scale from never (1) to always (5). The restrained eating score is obtained by dividing the total items’ score by the total number of items. Higher scores indicate a greater level of restrained eating (Cronbach’s α = 0.911).

#### ORTO-15

The ORTO-15 is a self-reported measure used to measure orthorexia nervosa [38]. It is composed of 15 multiple-choice items and is validated in Lebanon [31]. The answers are calculated on a 4-point Likert scale from never to always. In this research, a cut-off value of less than 40 was considered to predict the tendency of orthorexia nervosa. Lower scores indicate a higher tendency for orthorexia nervosa (Cronbach’s α = 0.791)*.*

#### DOS

The Dusseldorf Orthorexia Scale (DOS) [39], validated in Lebanon [40, 41], is a 10-item self-reported questionnaire to measure orthorexic eating behavior. Answers are scored on a 4-point Likert scale from 1 (this does not apply to me) to 4 (this applies to me), with higher points indicating a more pronounced orthorexic behavior. Scores between 25 and 29 indicate probable ON tendencies, whereas scores of 30 or more indicate the presence of ON [42]. The scale reliability in the current dataset was high (Cronbach’s α = 0.879).

#### TOS

The Teruel Orthorexia Scale (TOS) [43], validated in Lebanon [30, 44], is a relatively new scale consisting of 17 items rated on a 4-point Likert scale from 0 (completely disagree) to 3 (completely agree). This scale yields two subscale scores: healthy orthorexia (HeOr) and orthorexia nervosa (OrNe). The OrNe score was used in this study, where higher scores indicate higher orthorexia nervosa. This subscale showed very good reliability (Cronbach’s α OrNe = 0.852).

Three different scales were used to assess orthorexia nervosa because different interpretations of results and mixed ones were obtained following the use of different tools used for the screening of OrNe tendencies and behaviors [40].

### Statistical analyses

Data were analyzed on SPSS software version 23. Missing data was not replaced since it constituted < 5% of the total database. To confirm the psychometric properties of the body dissatisfaction subscale, a factor analysis was conducted on the scale’s items. Models’ adequacy was confirmed via the Kaiser–Meyer–Olkin (KMO) and Bartlett’s test of sphericity. Factors with Eigen values > 1 were retained. The normality of distribution of restrained eating, binge eating, ORTO-15, TOS OrNe, and DOS scores was confirmed by calculating the skewness and kurtosis; values for asymmetry and kurtosis between − 2 and + 2 are considered acceptable to show normal univariate distribution [45]. These conditions consolidate the assumptions of normality in sample sizes larger than 300 [46]. The Chi-square test compared categorical variables, and the ANOVA tested the differences among the means of three groups. Multivariable analysis of covariance (MANCOVA) was used to compare multiple measures among the three cluster groups, considering potential confounding variables, including age, monthly income, body mass index, marital status, education level, and body dissatisfaction. Furthermore, Cronbach’s alpha values were recorded for reliability analysis for all the scales. A *p* < 0.05 was considered significant.

## Results

### Sociodemographic and other characteristics of the participants

Table 1 displays the sociodemographic and other characteristics of the participants. The mean age of the women was 40.24 ± 7.51 years in the premenopause group, 43.64 ± 6.86 years in the perimenopause group, and 56.18 ± 9.66 years in the postmenopause group.

**Table 1:** Sociodemographic and other characteristics of the participants (N = 1001)

Variable	Premenopause (N = 395; 39.5%)	Perimenopause (N = 328; 32.8%)	Postmenopause (N = 278; 27.8%)	*p*
Age (in years)	40.24 ± 7.51	43.64 ± 6.86	56.18 ± 9.66	** < 0.001**
Marital status				0.225
Single/divorced/widowed	140 (42.8%)	106 (32.4%)	81 (24.8%)	
Married	255 (37.8%)	222 (32.9%)	197 (29.2%)	
Education level				** < 0.001**
Primary	18 (15.1%)	27 (22.7%)	74 (62.2%)	
Complementary	59 (29.8%)	62 (31.3%)	77 (38.9%)	
Secondary	80 (34.0%)	98 (41.7%)	57 (24.3%)	
University	238 (53.0%)	141 (31.4%)	70 (15.6%)	
Family monthly income				**0.03**
No income	114 (37.6%)	89 (29.4%)	100 (33.0%)	
< 1000 USD	117 (35.3%)	117 (35.3%)	97 (29.3%)	
1000–2000 USD	121 (45.5%)	86 (32.3%)	59 (22.2%)	
> 2000 USD	43 (42.6%)	36 (35.6%)	22 (21.8%)	
BMI categories				** < 0.001**
Normal (< 25 kg/m^2^)	226 (47.5%)	154 (32.4%)	96 (20.2%)	
Overweight (25–29.99 kg/m^2^)	130 (35.2%)	122 (33.1%)	117 (31.7%)	
Obese (≥ 30 kg/m^2^)	36 (24.3%)	51 (34.5%)	61 (41.2%)	
Body Mass Index (kg/m^2^)	24.76 ± 4.00	26.65 ± 13.66	26.98 ± 4.67	** < 0.001**
Body dissatisfaction score	10.68 ± 7.23	12.59 ± 7.57	12.63 ± 7.70	** < 0.001**
Restrained eating score	1.87 ± 0.98	1.90 ± 0.98	1.87 ± 0.87	0.968
ORTO-15 orthorexia nervosa score	26.56 ± 8.11	26.06 ± 8.08	24.12 ± 8.24	**0.001**
TOS orthorexia nervosa score	8.07 ± 5.63	7.92 ± 5.68	8.38 ± 6.08	0.725
DOS orthorexia nervosa score	22.07 ± 7.61	20.63 ± 7.19	21.89 ± 7.15	**0.033**
Binge eating score	10.94 ± 9.05	12.61 ± 9.73	11.07 ± 9.38	**0.047**

Numbers in bold indicate significant p-values

Bonferroni post hoc analysis: age (premenopause vs perimenopause *p* < 0.001; premenopauase vs postmenopause *p* < 0.001; peri vs postmenopauase *p* < 0.001); BMI (premenopause vs perimenopause *p* = 0.01; premenopauase vs postmenopause *p* = 0.003); body dissatisfaction (premenopause vs perimenopause *p* = 0.002; premenopauase vs postmenopause *p* = 0.003); ORTO-15 (premenopauase vs postmenopause *p* < 0.001; peri vs postmenopauase *p* = 0.011); TOS healthy orthorexia (premenopause vs perimenopause *p* = 0.008); DOS (premenopause vs perimenopause *p* = 0.027)

### Factor analysis of the body dissatisfaction subscale of the eating disorder inventory‑second version

All items were extracted and yielded a two-factor solution explaining 61.34% of the variance of the model (Table 2).

**Table 2:** Factor analyses of the body dissatisfaction scale

Item	Factor 1	Factor 2	H2 communalities
4. I feel satisfied with the shape of my body	0.818		0.683
3. I think that my stomach is just the right size	0.761		0.564
7. I think that my thighs are just the right size	0.741		0.566
5. I like the shape of my buttocks	0.741		0.521
9. I think that my hips are just the right size	0.725		0.536
6. I think my hips are too big		0.866	0.711
2. I think that my thighs are too large		0.859	0.774
I think by buttocks are too large		0.823	0.632
1. I think that my stomach is too big		0.634	0.534
Explained variance	39.77	21.57	
Cronbach’s alpha	0.815	0.815	

KMO = 0.775; Bartlett test of sphericity *p*  < 0.001; total explained variance = 61.34%

**Fig. 1:**
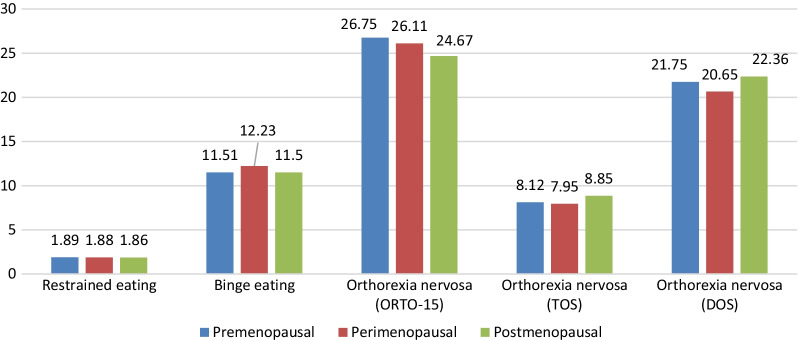
Mean disordered eating scores after adjustment over all covariates. Restrained eating (*p* = 0.965), Binge eating (*p* = 0.479), ORTO-15 orthorexia nervosa (*p* = 0.037), TOS orthorexia nervosa (*p* = 0.266), DOS orthorexia nervosa (*p* = 0.026). In the global model, the covariates were age, monthly income, body mass index, marital status, education level

Significantly high percentages of women with a primary educational level (62.2%) and no income (33.0%) were found in the postmenopause group. Postmenopausal women had a higher mean BMI compared to the two other groups. After adjusting for all covariates (age, marital status, education level, family monthly income, and BMI), more orthorexia nervosa tendencies (lower mean ORTO-15 scores) and higher orthorexia nervosa mean scores (as measured by the DOS) were found in postmenopausal women. Finally, no significant difference was found between the three groups regarding restrained eating, binge eating, and orthorexia nervosa (as measured by the TOS) (Fig. 1).

### Multivariable analysis

The MANCOVA analysis was performed, considering the eating disorders scales scores as the dependent variables and the pre, peri, and postmenopausal phases as the independent variables, after adjusting for age, monthly income, BMI, marital status, education level, and body dissatisfaction. Postmenopause was significantly associated with more orthorexia nervosa tendencies (lower ORTO-15 scores) than premenopause (β =  −  1.87; *p* = 0.022). Perimenopause was associated with more binge eating (β = 1.56; *p* = 0.031), and less orthorexia nervosa (as measured by the DOS) than premenopause, with this association tending to significance (β = − 1.10; *p* = 0.051) (Table 3). Furthermore, higher body dissatisfaction was significantly associated with higher restrained eating (β = 0.02; *p* < 0.001), binge eating (β = 0.48; *p* < 0.001), and orthorexia nervosa as measured by TOS (β = 0.08; *p* = 0.002) (Additional file 1: Table S1).

**Table 3:** Multivariate analysis of covariance (MANCOVA)

	Crude results				Adjusted results			
	Beta	*p*	Confidence interval		Beta	*p*	Confidence interval	
			Lower	Upper			Lower	Upper
Restrained eating score								
Perimenopause versus premenopause*	0.03	0.729	− 0.12	0.17	− 0.01	0.899	− 0.20	0.18
Postmenopause versus premenopause*	0.001	0.984	− 0.15	0.15	0.01	0.872	− 0.13	0.15
Binge eating score								
Perimenopause versus premenopause*	1.68	**0.017**	0.30	3.05	1.56	**0.031**	0.14	2.97
Postmenopause versus premenopause*	0.14	0.854	− 1.31	1.58	0.42	0.659	− 1.45	2.30
Orthorexia nervosa (ORTO-15 scale)								
Perimenopause versus postmenopause*	− 0.50	0.413	− 1.70	0.70	− 0.34	0.580	− 1.55	0.87
Postmenopause versus premenopause*	− 2.44	** < 0.001**	− 3.70	− 1.18	− 1.87	**0.022**	− 3.47	− 0.27
Orthorexia nervosa (TOS scale)								
Perimenopause versus premenopause*	− 0.15	0.733	− 0.99	0.70	− 0.08	0.864	− 0.94	0.79
Postmenopause versus premenopause*	0.31	0.488	− 0.57	1.20	0.81	0.165	− 0.34	1.96
Orthorexia nervosa (DOS scale)								
Perimenopause versus premenopause*	− 1.43	**0.009**	− 2.51	− 0.36	− 1.10	0.051	− 2.20	0.01
Postmenopause versus premenopause*	− 0.18	0.751	− 1.31	0.95	0.66	0.374	− 0.80	2.13

*Reference group; Numbers in bold indicate significant *p* values. *Note*: In the adjusted model, the covariates were age, monthly income, body mass index, marital status, education level

The results of the logistic regressions, taking the dichotomous disordered eating variables (presence vs absence) as dependent variables, showed that perimenopause was significantly associated with higher odds of binge eating compared to premenopause (aOR = 1.45; *p* = 0.04) (Table 4).

**Table 4:** Multivariate analyses: Logistic regressions taking the dichotomous scores of the eating disorders scales as dependent variables

	Crude results				Adjusted results			
	*p*	aOR	Confidence interval		*p*	aOR	Confidence interval	
			Lower	Upper			Lower	Upper
Presence versus absence of restrained eating								
Perimenopause versus premenopause*	0.758	0.96	0.71	1.28	0.825	0.97	0.71	1.31
Postmenopause versus premenopause*	0.371	0.87	0.64	1.18	0.707	0.92	0.61	1.39
Presence versus absence of Binge eating score								
Perimenopause versus premenopause*	**0.005**	1.62	1.16	2.26	**0.04**	1.45	1.02	2.06
Postmenopause versus premenopause*	0.601	1.10	0.77	1.59	0.812	0.94	0.58	1.53
Presence versus absence of Orthorexia nervosa (ORTO-15 scale)								
Perimenopause versus postmenopause*	0.573	1.26	0.56	2.85	0.549	1.29	0.56	3.01
Postmenopause versus premenopause*	0.233	1.79	0.69	4.68	0.269	1.97	0.59	6.58
Presence versus absence of Orthorexia nervosa (TOS scale)								
Perimenopause versus premenopause*	0.272	0.85	0.63	1.14	0.300	0.85	0.62	1.16
Postmenopause versus premenopause*	0.938	1.01	0.74	1.38	0.524	1.14	0.76	1.71
Presence versus absence of Orthorexia nervosa (DOS scale)								
Perimenopause versus premenopause*	0.261	0.84	0.61	1.14	0.513	0.90	0.65	1.24
Postmenopause versus premenopause*	0.513	0.90	0.65	1.24	0.908	0.98	0.63	1.50

*Reference group; Numbers in bold indicate significant *p* values. *Note*: In the adjusted model, the covariates were age, monthly income, body mass index, marital status, education level

## Discussion

The objective of our study was to assess whether menopause phases are associated with disordered eating (restrained eating, binge eating, and orthorexia nervosa) after adjustment over sociodemographic variables and body dissatisfaction in a sample of middle-aged Lebanese women. Our results suggest that women in postmenopause had more orthorexia nervosa tendencies, while those in perimenopause had more binge eating and less orthorexia nervosa.

### Menopausal phases and binge eating

Our results showed that binge eating was significantly higher in the perimenopause group. Consistently, previous work suggests that perimenopausal women had a higher prevalence of disordered eating and a significantly higher self-rating score of “feeling fat” than premenopausal women [31]. The transition to menopause consists of several complex physical, hormonal, mental, and social changes occurring in women and making them vulnerable to psychological distress [31]. It is known that sleep disturbances caused by vasomotor symptoms are frequent among women in perimenopause [32], and these disturbances can lead to eating disorders, mostly binge eating [7], explaining binge eating tendencies in our sample.

### Menopausal phases and restrained eating

No remarkable differences were observed in our study for restrained eating between pre, peri, and postmenopausal women. However, a study showed that 15.7% of the total sample manifested clinically significant scores on restrained eating, and postmenopausal women had notably more restrained eating than premenopausal women [9]. Generally, the changes in body fat distribution due to menopause or the increase in BMI in middle-aged women are considered to impact appearance negatively [9], explaining why women are more prone to restrained eating in postmenopause. The literature showed that BMI and restrained eating are positively correlated [9]; in our sample, women in all three menopausal phases had a comparable BMI, which can explain the absence of difference between the three groups for restrained eating.

### Menopausal phases and orthorexia nervosa

Women in the postmenopausal phase are more prone to orthorexia nervosa (as measured by ORTO-15) compared to women in premenopause. In addition, perimenopausal women have lower tendencies to orthorexia nervosa. No directly comparable study was found in the literature, and the results were conflicting regarding the association between menopausal phases and disordered eating. While a study found no differences in eating disorders among pre, peri, and postmenopausal women [4], another study could demonstrate that perimenopausal women are at higher risk than premenopausal women of developing binge-related eating disorders rather than restrictive types [47]. Furthermore, women in postmenopause reported higher restrained eating and dietary disinhibition than premenopausal women [9]. During perimenopause, women are more prone to develop binge-eating disorders, which explains lower tendencies to orthorexia nervosa.

### Body dissatisfaction and eating disorders

In our sample, higher body dissatisfaction was significantly associated with higher restrained eating, binge eating, and orthorexia nervosa, consistent with previous findings showing that body dissatisfaction predicts the development of eating disorders [48]. A study among a sample of overweight and obese adolescent girls reported that those who were the most pleased with their bodies expressed 85% lower odds of starting to binge frequently than their less satisfied peers [49]. Other findings suggest that body image and self-esteem directly affected restrained eating and that body dissatisfaction had a significant positive total effect on restrained eating and binge eating in women [50]. Consistently with our results, a study among a sample of Lebanese women showed that higher body dissatisfaction, higher restrained eating, and higher binge eating were markedly related to higher levels of orthorexia tendencies and behaviors [33]. Self-esteem happens to be an essential factor that can affect eating disorders [18, 48]. People with high self-esteem care less about their body image and are less prone to developing binge eating, orthorexia nervosa, or restrained eating.

### Clinical implications

Our study showed that menopausal stages are associated with some disordered eating behaviors (binge eating and orthorexia nervosa) among middle-aged women. Those results may serve as a first step towards spreading awareness among women within this age group regarding eating attitudes. Moreover, healthcare professionals should screen for the presence of disordered eating during those women’s routine visits to the clinics.

### Limitations

This study has some limitations. The use of a self-report questionnaire clinician-administered interviews (for an accurate clinical diagnosis and functional impairments assessment) can only provide an estimate of symptoms severity and lead to recall bias. BMI was calculated based on self-reported weight and height. A confounding bias might also exist since the questionnaire did not include all the variables known to be associated with disordered eating (i.e. current use of menopausal hormone therapy, presence of comorbidities (hypertension, diabetes mellitus, etc.), number of family members in the house, which is important for the monthly expenses, physical activity, climacteric symptoms as well as physical and psychological changes that postmenopausal women experience compared to pre- and peri-menopause women may affect every measure in this study). Another important variable that was not assessed is the climacteric symptoms, as it may have given us important information on whether climacteric symptoms can be associated with eating disorders or not. A selection bias is present because of the refusal rate. The sample was not selected randomly; therefore, the results cannot be generalized to the whole population. This study does not assess prior mental health history or eating disorder symptoms. So, it is unknown if participants developed their eating disorder-related symptoms before the menopause transition or following it. This study is cross-sectional; thus, it does not allow to infer the causality of the relationship between menopausal phases and eating disorders; Further longitudinal studies are necessary to elucidate whether existing disordered eating symptoms become more pronounced in menopause or symptoms of eating disorders develop because of menopause. Despite all these limitations, we believe that our results are of value since they corroborate those of previous international findings.

## Conclusion

This study evaluated the association between menopause phases and abnormal eating habits (restrained eating, binge eating, and orthorexia nervosa) in a sample of middle-aged Lebanese women. Our findings indicate that postmenopause was significantly associated with more orthorexia nervosa tendencies (but not binge or restrained eating) than premenopause. Further studies exploring processes such as the potential interaction of estrogen and progesterone are necessary to confirm our results.

## Supplementary Information

**Additional file 1.** **Supplementary Table 1.** Multivariate analysis of covariance (MANCOVA).

## Data Availability

The datasets generated and/or analysed during the current study are not publicly available due to the ethics committee rules and regulations but are available from the corresponding author on reasonable request.

## Abbreviations

ED: Eating disorders
DSM-4: Diagnostic and Statistical Manual of Mental Disorders-4th edition
EDI-2: Eating Disorder Inventory‑second version
BES: Binge Eating Scale
DOS: Dusseldorf Orthorexia Scale
TOS: Teruel Orthorexia Scale
MANOCVA: Multivariable analysis of covariance
