# Supplementary material for: Eating disorders and their relationship with menopausal phases among a sample of middle-aged Lebanese women
Source: BMC Womens Health. 2022 May 10;22:153. doi: 10.1186/s12905-022-01738-6 (PMC9092875; doi:10.1186/s12905-022-01738-6)
Supplement: Supplementary file 1 — Additional file 1. Supplementary Table 1. Multivariate analysis of covariance (MANCOVA). [file 12905_2022_1738_MOESM1_ESM.docx]

| **Supplementary Table 1. Multivariate analysis of covariance (MANCOVA)** | | | | |
| --- | --- | --- | --- | --- |
|  | **Beta** | ***p*** | **Confidence interval** | |
|  |  |  | **Lower** | **Upper** |
| **Restrained eating score** | | | | |
| Body dissatisfaction | 0.02 | **<0.001** | 0.01 | 0.03 |
| Complementary vs primary* education level | 0.39 | **<0.001** | 0.18 | 0.61 |
| Secondary vs primary* education level | 0.44 | **<0.001** | 0.22 | 0.66 |
| University vs primary* education level | 0.44 | **<0.001** | 0.21 | 0.66 |
| Intermediate monthly salary 1000-2000 USD vs no income* | 0.27 | **0.002** | 0.10 | 0.43 |
| High monthly salary >2000 USD vs no income* | 0.39 | **0.001** | 0.16 | 0.62 |
| Perimenopause vs premenopause* | -0.01 | 0.912 | -0.15 | 0.13 |
| Postmenopause vs premenopause* | -0.03 | 0.791 | -0.21 | 0.16 |
| **Binge eating score** |  |  |  |  |
| Body Mass Index | 0.09 | **0.005** | 0.03 | 0.16 |
| Body dissatisfaction | 0.48 | **<0.001** | 0.41 | 0.56 |
| Perimenopause vs premenopause* | 0.72 | 0.278 | -0.58 | 2.02 |
| Postmenopause vs premenopause* | -0.01 | 0.994 | -1.72 | 1.71 |
| **Orthorexia nervosa (ORTO-15 scale)** |  |  |  |  |
| Complementary vs primary* education level | 1.84 | 0.051 | -0.01 | 3.69 |
| Secondary vs primary* education level | 2.19 | **0.021** | 0.34 | 4.04 |
| University vs primary* education level | 2.50 | **0.011** | 0.58 | 4.42 |
| Perimenopause vs postmenopause* | -0.64 | 0.294 | -1.84 | 0.56 |
| Postmenopause vs premenopause* | -2.08 | **0.01** | -3.67 | -0.49 |
| **Orthorexia nervosa (TOS scale)** |  |  |  |  |
| Body Mass Index | -0.06 | **0.011** | -0.10 | -0.01 |
| Body dissatisfaction | 0.08 | **0.002** | 0.03 | 0.13 |
| Perimenopause vs premenopause* | -0.17 | 0.698 | -1.04 | 0.70 |
| Postmenopause vs premenopause* | 0.73 | 0.212 | -0.42 | 1.88 |
| **Orthorexia nervosa (DOS scale)** |  |  |  |  |
| Body Mass Index | -0.08 | **0.004** | -0.14 | -0.03 |
| Perimenopause vs premenopause* | -1.10 | 0.051 | -2.20 | 0.01 |
| Postmenopause vs premenopause* | 0.62 | 0.409 | -0.84 | 2.07 |
| Note: In the global model, the covariates were age, monthly income, body mass index, marital status, education level and body dissatisfaction.  *Reference group; Numbers in bold indicate significant p-values. | | | | |
